# Experimental and Numerical Evaluation of Unsaturated Polyester Polymer Concrete for Highway Pavement Repair and Surface Rehabilitation

**DOI:** 10.3390/polym18101217

**Published:** 2026-05-16

**Authors:** Bircan Arslannur, Muhammed A. Ozdemir, Ferit Cakir

**Affiliations:** 1 Department of Civil Engineering, Igdir University, Igdir 76000, Türkiye; barslannur@igdir.edu.tr (B.A.); m.alperen.ozdemir@igdir.edu.tr (M.A.O.); 2 Department of Civil Engineering, Gebze Technical University, Kocaeli 41400, Türkiye

**Keywords:** polymer concrete (PC), unsaturated polyester resin, partial-depth repair, finite element analysis, compressive strength, rapid curing materials

## Abstract

Pavement repair has become an increasingly time-critical operation as traffic volumes grow and lane-closure windows shrink. This has driven demand for materials that gain full structural strength quickly, reopen to traffic within hours, and hold up longer than conventional patches. This study evaluates polymer concrete (PC), a thermosetting resin-bound aggregate system, through combined laboratory characterization and three-dimensional finite element analysis. Compressive strength, splitting tensile strength, unit weight, and apparent porosity were measured at 1, 3, 7, and 28 days of curing. PC reached 85.97 MPa in compression and 7.63 MPa in tension by day three, with near-zero porosity (0.15%) maintained throughout. These three-day values were used directly as material inputs in the three-dimensional finite element analysis (FEA), reflecting the early traffic reopening scenario that defines rapid repair practice. Structural performance was assessed through 36 static analyses in ANSYS 2024 R2, covering flexible (Hot Mix Asphalt, HMA) and rigid (Jointed Plain Concrete Pavement, JPCP) pavement types, three patch sizes (250 × 250 mm, 500 × 500 mm, and 1000 × 1000 mm), and nine load scenarios per configuration. Safety factors (SF) against internal cracking, interfacial debonding, and compressive failure were computed for both PC and traditional patches. PC consistently outperformed HMA and Portland cement concrete patches across all metrics. On rigid pavements, interfacial safety factors exceeded 22.0, confirming that standard surface preparation is sufficient. On flexible pavements, adopting 0.78 MPa as a conservative lower-bound estimate of PC-HMA interfacial bond strength, five scenarios exhibit debonding risk (250-C, 500-C, 500-D, 1000-C, and 1000-D; SF = 0.47–0.99), while the remaining four show high interfacial risk (SF = 1.11–1.30); primer application and mechanical scarification are required for all PC repairs on flexible pavements regardless of patch geometry. Taken together, the experimental and numerical evidence positions PC as a credible, high-performance option for highway repair.

## 1. Introduction

Highway pavements work hard. They absorb traffic loading day and night, expand and contract with the seasons, and take repeated hits from de-icing chemicals and moisture that gradually weaken their structure from within. The cumulative damage is familiar: cracks, spalls, potholes, and the kind of structural deterioration that keeps maintenance crews busy and drives up life-cycle costs. Conventional cement-based concretes carry well-known limitations, particularly low tensile strength, shrinkage cracking, moisture susceptibility, and chemical vulnerability, which contribute to premature repair failures and reduced durability [1,2]. These shortcomings have kept the search for better repair materials open for decades.

Polymer concrete (PC), a composite in which mineral aggregates are bound entirely by synthetic polymer resin rather than Portland cement, has attracted growing interest as a structural repair material, largely because its physical, mechanical, and fracture properties measurably exceed those of ordinary Portland cement concrete [3,4,5]. Because the hardening reaction is polymerization rather than cement hydration, PC gains strength within hours, no extended wet-curing required. Early studies by Ohama and Demura (1982) [6] showed that polyester-based PC accumulates most of its compressive strength within the first day or two, with properties stabilizing soon after. More recent experimental evidence confirms that PCs may achieve more than 80% of their long-term mechanical capacity within the first three to seven days [7,8]. For road agencies working inside tight lane-closure windows, that rate of strength gain matters.

Beyond rapid strength gain, PCs exhibit high mechanical performance, including superior compressive, tensile, and shear resistance, attributes linked to their dense polymer matrix and strong aggregate-binder interaction [1]. Reported compressive strengths commonly exceed those of conventional cementitious materials, while tensile capacity and surface hardness support structural load transfer and abrasion resistance required for pavement overlays. The absence of interconnected capillary pores further contributes to low permeability, high chemical resistance, and long-term durability under freeze–thaw cycling, chloride exposure, fuel spills, and other roadway service conditions [2,9]. PCs also demonstrate strong adhesion to existing asphalt and concrete substrates, making them suitable for partial-depth repairs, pothole patching, and surface rehabilitation [10]. Despite these advantages, large-scale implementation in pavement engineering remains limited. Existing research has primarily focused on material characterization, curing mechanisms, or polymer chemistry rather than transportation-specific considerations such as field constructability, compatibility with existing pavement layers, and performance under continuous traffic loading. Furthermore, standardized testing and design guidelines for pavement applications are still developing, creating uncertainties for transportation agencies and practitioners [11]. Accordingly, continued experimental research is needed to establish performance benchmarks, durability expectations, and application suitability of PC for highway pavement rehabilitation. In particular, studies evaluating compressive and tensile behavior, porosity, density, and early-age curing characteristics can provide essential material-level evidence to support engineering decision-making, specification development, and rapid-repair strategies.

Despite growing interest in rapid rehabilitation, PC for pavement repair has received comparatively little attention in the literature. Unlike previous investigations that focused primarily on general structural use, resin chemistry, or laboratory curing optimization, the present study evaluates mechanical performance parameters directly relevant to field implementation, including compressive and tensile capacity, density, and porosity, all of which govern traffic reopening time, durability, and long-term serviceability. Motivated by the increasing severity of pavement distress and the performance-based demands of international regulatory frameworks, this work provides experimentally validated benchmarks for plain (non-fiber), low-porosity PC mixtures in a pavement repair context, offering data that practitioners and specification writers can apply directly. The core finding, structural-level strength within three days, has direct implications for work-zone scheduling, closure duration, and long-term patch reliability on heavily trafficked routes.

Highway pavements experience continuous structural and functional degradation throughout their service life due to a combination of mechanical, environmental, and operational factors. Increasing freight transportation, rising axle loads, higher traffic density, and expanding urban mobility demands place significant stress on pavement systems. As a result, surface and subsurface distress, including fatigue cracking, potholes, rutting, raveling, joint spalling, and faulting, emerges earlier and progresses faster than originally anticipated in design stages. These deteriorations not only compromise ride quality and user comfort but also increase vehicle operating costs, fuel consumption, noise, emissions, and crash probability, particularly on high-speed, multilane roadway corridors.

Recognizing the safety and operational risks associated with deteriorated pavements, international transportation authorities explicitly emphasize early detection and timely rehabilitation. The Federal Highway Administration (FHWA) identifies pavement distress as a leading contributor to roadway departures and work-zone crashes, urging agencies to minimize the duration and frequency of lane closures by prioritizing rapid, high-performance repair materials [12]. Similarly, the AASHTO Pavement Design and Management Guidelines highlight the need for repair systems capable of resisting heavy truck loading, moisture infiltration, and freeze–thaw exposure, while also enabling immediate or near-immediate reopening to traffic [13,14,15]. In Europe, EN 1504 parts 1 to 10 structural repair standards and EN 13108 asphalt specifications require rehabilitation materials to demonstrate high mechanical strength, low permeability, dimensional stability, and long-term durability under environmental and chemical exposure [16,17,18].

Traditional pavement repair approaches, such as hot mix asphalt patching, cement-based concrete overlays, cold patch materials, and surface slurry seals, remain widely used due to familiarity, cost accessibility, and established practices. However, these methods often present performance limitations. Asphalt-based repairs may soften under high temperatures, deform under concentrated loading, or fail prematurely under moisture intrusion. Portland cement-based mixes, while structurally reliable, typically require prolonged curing periods before traffic reopening, during which hydration, shrinkage cracking, and debonding may occur. Moreover, many conventional materials possess relatively high porosity, allowing water and chloride penetration that accelerates subsurface deterioration. These limitations contribute to short service life, repeated maintenance cycles, increased life-cycle costs, and prolonged roadway disruptions, conditions incompatible with modern transportation system performance expectations.

Contemporary traffic engineering philosophy emphasizes resilience, mobility preservation, and safety-driven maintenance planning. In dense urban networks, even short repair-related closures create substantial congestion, delay emergency response vehicles, disrupt freight movement, and increase crash exposure near work zones. As a result, international guidelines increasingly advocate for the adoption of rapid-setting, high-strength, durable, and low-permeability repair materials capable of restoring structural capacity within hours rather than days or weeks. FHWA’s Rapid Renewal Program and AASHTO’s Transportation Asset Management directives both encourage the evaluation of alternative material technologies to achieve faster, longer-lasting interventions [13,19]. Likewise, the World Road Association (PIARC) recommends integrating new-generation composites into pavement rehabilitation strategies to reduce maintenance frequency and environmental impact [20].

For this reason, the increasing severity of pavement distress and the performance-based expectations set by international regulatory frameworks reinforce the need to explore and experimentally validate advanced repair materials, such as PC, that may fundamentally improve the efficiency, durability, and sustainability of modern pavement rehabilitation practices.

To place the present results in context, we compare them quantitatively with the recent UPR-PC literature. Reviews of polymer concrete report compressive strengths typically in the range of 70–120 MPa, with peak values strongly dependent on resin content (10–20% by weight) and on aggregate packing density [1,21,22]. Plain UPR-PC mixtures previously tested by our group reached 80–95 MPa in compression and 6–9 MPa in splitting tension, while polyester polyurethane concrete used as a steel bridge deck overlay reaches 75.3 MPa compressive and 8.4 MPa splitting tensile strength [23,24]. The 3-day values obtained here, 85.97 MPa in compression and 7.63 MPa in splitting tension, fall within these ranges. The primary distinguishing factor of the present mixture is its accelerated development; the target performance is achieved within three days, as opposed to the conventionally reported 28-day period. The apparent porosity of 0.15% is attributed to two primary design parameters, specifically a multi-fraction natural river-bed aggregate skeleton (35% in 0–1 mm, 40% in 1–3 mm, and 25% in 3–5 mm) and a 13% resin content.

This work distinguishes itself from prior literature through four primary contributions. First, it introduces an early-age characterization tied to rapid-reopening practices using a field-replicable, plain mixture. While most studies on unsaturated polyester resin polymer concrete (UPR-PC) report 28-day properties for fiber-reinforced or modified variants, this research measures mechanical properties at 1, 3, 7, and 28 days, explicitly designating the 3-day values as the design-critical reference. The formulation is deliberately kept simple; utilizing commercial UPR resin, an MEKP catalyst, a cobalt accelerator, and graded natural river-bed aggregate; allowing highway agencies to reproduce it without specialty additives. Building on this experimental foundation, the study establishes a direct experimental-to-numerical pipeline. Unlike earlier pavement-repair finite element analysis (FEA) studies that typically adopted properties from secondary sources [25], this work directly utilizes its own measured 3-day strength, modulus, and Poisson’s ratio as FEA inputs. Furthermore, a dual-pavement parametric framework is introduced to provide practitioner-actionable design rules. By evaluating the same UPR-PC mixture on both HMA and JPCP host pavements across a 36-case parametric matrix under four critical loading positions, it offers the first direct cross-pavement comparison of safety factors for plain UPR-PC. Comparing these factors against EN 1504-3 [17] and ACI 548.1R [11] thresholds yields explicit, geometry-dependent recommendations. Finally, the study presents a transparent interface-analysis methodology. This integrated approach combines a Multi-Point Constraint contact formulation, a localized 1 mm mesh refinement at the patch-substrate boundary, and a path-based stress extraction taken 2 mm from the singularity (justified by a Saint-Venant decay argument). These numerical outputs are explicitly compared against substrate-specific bond capacities (0.78 MPa for PC-HMA and 2.82 MPa for PC-JPCP), enabling a direct interfacial debonding risk classification for plain UPR-PC patches under traffic loading.

## 2. Materials and Methods

### 2.1. Materials

PC mixtures were produced using an unsaturated polyester resin as the primary binder, selected due to its rapid curing behavior, strong aggregate adhesion, and suitability for transportation infrastructure applications. The technical characteristics of the resin are summarized in Table 1.

To initiate and regulate polymerization, methyl ethyl ketone peroxide (MEKP) was used as the curing catalyst, while cobalt naphthenate served as the accelerator. Their key properties are listed in Table 2 and Table 3, respectively.

Natural river-bed aggregates of mixed mineralogical composition (predominantly quartz with carbonate constituents typical of natural fluvial deposits, as confirmed by XRD and EDS analyses in Section 4.1) were selected due to their hardness, abrasion resistance, and established use in pavement structures. Three gradation fractions were combined to ensure dense packing and reduced void formation. Aggregates were oven-dried at 105 ± 5 °C for 24 h before mixing to prevent moisture interference with polymerization. Aggregate properties are provided in Table 4.

### 2.2. Mixture Design and Proportioning

A preliminary trial-mix program was conducted to identify an optimum balance between workability, polymerization rate, and coating efficiency. MEKP contents of 0.90%, 1.00%, 1.25%, and 1.50% (by weight of resin) were evaluated. The 1.25% dosage produced the most uniform curing without premature gelation and was therefore adopted. The cobalt accelerator dosage was maintained at 1.50% of resin mass. The final mixture proportions used throughout the study are presented in Table 5.

### 2.3. Mixing and Casting Procedure

Mixing was carried out in a stainless-steel rotary mixer. Aggregates were first dry-blended for 60 s to ensure uniform gradation. The polyester resin and cobalt accelerator were then added and mixed for 90 s, allowing complete aggregate coating. Finally, MEKP was introduced and mixed for approximately 45 s to activate polymerization while maintaining workable consistency. No water or cementitious components were used. Although the neat resin exhibits a gel time of approximately 5.3 min, the addition of mineral aggregates effectively extends the practical working window by acting as a thermal sink during the exothermic polymerization reaction. This behavior is consistent with established practice for polyester-based PC systems, where aggregate-loaded mixtures provide sufficient open time for mixing, casting, and compaction operations [6]. The total elapsed time from MEKP introduction to completion of compaction was maintained well within this extended working window throughout the experimental program. Cylindrical molds (Ø100 × 200 mm) were prepared for compressive and splitting tensile strength testing, while 50 × 50 × 50 mm cubic molds were used for density and porosity characterization. Specimens were compacted on a vibration table to remove entrapped air and ensure matrix homogeneity. Demolding occurred 2–3 h after casting once sufficient rigidity was achieved.

### 2.4. Curing Protocol

All specimens were stored at 23 ± 2 °C and 50 ± 5% relative humidity until testing. Because PC strength gain is governed by polymerization rather than hydration, no moist curing or water submersion was applied. Mechanical and physical tests were conducted at 1, 3, 7, and 28 days, time intervals representing critical pavement repair and traffic reopening decisions in transportation engineering practice.

### 2.5. Mechanical and Physical Testing

Compressive strength testing followed ASTM C39 procedures using a servo-controlled universal testing machine at a loading rate of 0.25 MPa/s [26]. Splitting tensile strength was determined using ASTM C496 with plywood bearing strips to prevent localized crushing [27]. For each age and test category, at least three specimens were evaluated. Density and apparent porosity were measured according to ASTM C642 using oven-dry, saturated, and submerged mass determinations [28]. These parameters were selected due to their relevance in predicting field durability, moisture susceptibility, and freeze–thaw performance of pavement repairs. The elastic modulus of the PC mixture was determined from the compressive stress–strain response recorded during the 3-day uniaxial compression tests, following the procedure outlined in ASTM C469 [29]. The secant modulus was calculated from the linear portion of the load-deformation curve between stress levels of 0.05*f*’c and 0.40*f*’c, using the average of three specimens. The resulting value of 21,247 MPa was adopted as the FEA input, consistent with the 3-day testing age used for all material capacity parameters in this study. Poisson’s ratio (*ν* = 0.18) was obtained from the simultaneous lateral strain measurements recorded during the same compression tests.

### 2.6. Finite Element Analysis

#### 2.6.1. Pavement Structure and Material Properties

**Figure 1:**
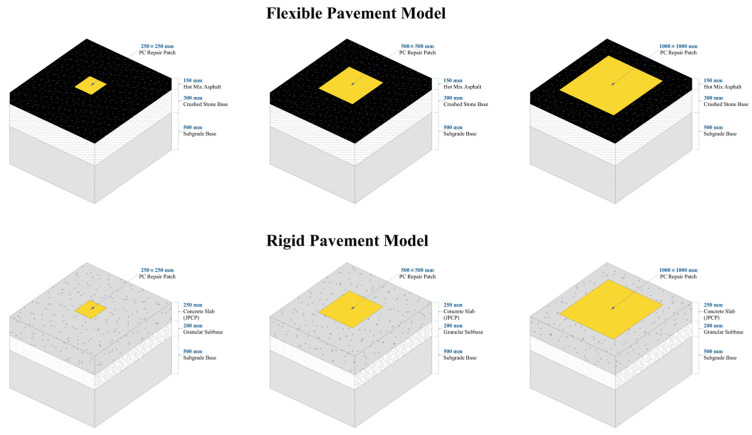
Schematic cross-sections of the flexible (HMA) and rigid (JPCP) pavement models used in the FEA study, showing layer thicknesses and material designations.

Pavement geometries were based on the design recommendations of the AASHTO Mechanistic-Empirical Pavement Design Guide [15]. Two structural configurations were modeled, as illustrated in Figure 1:

Rigid Pavement Model: Modeled as a Jointed Plain Concrete Pavement (JPCP), the slab features planar dimensions of 3.5 m × 4.5 m, selected in accordance with the typical joint spacing recommendations outlined in the Florida Department of Transportation (FDOT) guidelines [30]. The cross-section comprises a 250 mm (10 in.) concrete slab overlying a 200 mm (8 in.) granular subbase and a subgrade foundation.

Flexible Pavement Model: Consists of a 150 mm (6 in.) Hot Mix Asphalt (HMA) surface layer overlying a 300 mm (12 in.) crushed stone base and subgrade. Although flexible pavements typically comprise multiple asphalt lifts (e.g., wearing and binder courses), these were modeled as a single equivalent homogeneous layer under the assumption of a fully bonded monolithic condition, a simplification consistent with AASHTO design guidelines (Section 12.1.7 of [15]).

The repair geometry was standardized as a 50 mm Partial-Depth Repair (PDR) for both pavement types. This depth was selected to simulate realistic field practices where Polymer Concrete is utilized for high-performance surface restoration (e.g., correcting spalling or rutting) rather than as a bulk structural fill due to economic constraints. Accordingly, the model represents typical ‘mill-and-fill’ operations for flexible pavements and standard FHWA Class 2 repairs for rigid pavements, focusing the analysis on interface bond stresses, which constitute the primary failure mechanism for thin, high-modulus repairs.

To isolate the effect of pavement surface stiffness on the repair material performance, the foundation support conditions (Granular Base/Subbase and Subgrade properties) were kept identical for both rigid and flexible pavement models, representing a standard highway embankment on a stiff soil. Material properties for the pavement layers were selected based on the “Input Level 3” (default) recommendations provided in Section 11 of the AASHTO guide [15]. Since the standard specifies values in US Customary units (psi), these were converted to SI units (MPa) for the FEA simulation:

Bound Layers: The concrete slab modulus was defined as 27,500 MPa, which corresponds to the typical range for adequate intact Portland Cement Concrete (PCC) slabs (3 to 4 × 10^6^ psi) as presented in Table 11-5 of the AASHTO [15]. For the asphalt layer, a representative dynamic modulus of 3500 MPa (500,000 psi) was adopted, consistent with typical dense-graded HMA stiffness at standard service temperatures (70 °F) as per Table 11-3 of the AASHTO [15].

Unbound Layers: For the granular base and subbase, a resilient modulus of 275 MPa (40,000 psi) was selected, corresponding to the default value for A-1-a classification soils (Table 11-10 of the AASHTO [15]). The subgrade soil was modeled with a resilient modulus of 115 MPa (16,500 psi), representing a stiff A-2-4 soil (Table 11-10 of the AASHTO [15]).

The mechanical properties of the PC repair material (elastic modulus, compressive and tensile strength) were determined experimentally and are presented in detail in Section 3.1 and Section 3.2; these values are adopted as FEA input parameters consistent with the 3-day early traffic reopening scenario targeted in this study. A unit weight of 2110 kg/m^3^ was assigned, corresponding to the average experimental density of 2.11 g/cm^3^. Based on the 3-day compressive strength of the PC mixture (85.97 MPa), the elastic modulus and Poisson’s ratio were determined experimentally as 21,247 MPa and 0.18, respectively, from uniaxial compression tests following ASTM C469, as stated in Section 2.5. These 3-day values were adopted to represent early traffic reopening conditions, consistent with the rapid-repair application targeted in this study and ensure internal consistency between the FEA material inputs and the structural capacity values used in the safety factor assessment.

Table 6 summarizes the complete set of geometric dimensions and material parameters used in the FEA models.

The complex contact interactions and localized stress concentrations at the dowel-concrete interface, as extensively characterized by [31], were excluded from the scope of this study. Instead, a conservative ‘Worst-Case’ scenario was adopted by assuming zero load transfer efficiency (LTE = 0). This approach neglects the load-sharing capacity of dowel bars, thereby subjecting the repair patch to the maximum possible structural demand. In practice, LTE for well-maintained JPCP typically ranges from 50–80% [20]; LTE = 0 therefore represents a conservative lower bound, and the reported JPCP safety factors may overestimate the true structural demand on the repair patch. A parametric study varying LTE is identified as a priority for future investigation to quantify the degree of conservatism in the present results.

#### 2.6.2. Finite Element Model Setup

The application of finite element modeling to predict the complex mechanical responses of polymer concrete systems has been demonstrated as a highly effective computational approach in recent literature [32]. Consistent with these advanced computational frameworks, all finite element analyses in this study were performed using ANSYS 2024 R2 (Ansys, Inc., Canonsburg, PA, USA). The three-dimensional models were discretized using ten-node quadratic tetrahedral elements (SOLID187), which provide second-order displacement interpolation and are well-suited for capturing stress gradients in geometrically complex domains involving dissimilar material interfaces. The element order was set to Program Controlled, which defaults to quadratic formulation in ANSYS, ensuring accurate representation of bending-dominated and stress-concentration-sensitive regions without manual element selection.

Contact conditions at the interface between the repair patch and the existing pavement surface were defined using the Multi-Point Constraint (MPC) formulation, which enforces compatibility between contacting surfaces through constraint equations rather than penalty-based methods. This approach ensures full composite action between the patch and the substrate, representing an ideal adhesion condition consistent with recommended field practice, which typically involves surface scarification, cleaning, and primer application prior to polymer concrete placement. The MPC formulation was selected over standard bonded contact due to its superior numerical stability and more accurate stress transfer characteristics at the patch-pavement interface. This approach is widely adopted in numerical pavement repair studies [25,33] and provides a conservative upper-bound estimate of structural performance by eliminating the possibility of interfacial slip or delamination. It should be noted that the MPC contact formulation, while representing ideal bonded conditions, provides an upper-bound estimate of interfacial stress demand: under imperfect bonding conditions, such as absence of primer or inadequate surface preparation, interfacial slip would redistribute load over a wider area, reducing local stress concentrations. Thus, the interfacial safety factors reported herein represent conservative lower-bound estimates for adequately prepared surfaces, and the debonding risk identified for PC on HMA pavement specifically highlights that even under ideal contact assumptions, the interfacial stress demand approaches or exceeds the available bond strength, making primer application a physical necessity rather than merely a precautionary recommendation. The finite element model setup and boundary conditions were verified against published numerical pavement repair studies employing equivalent modeling strategies [25,33], both of which report good agreement between FEA predictions and experimental measurements using comparable mesh configurations and contact definitions. In the absence of a dedicated experimental validation campaign for the present models, confidence in the results is further supported by the internal consistency of the stress distributions: the maximum principal stress contours exhibit the expected patterns for each loading scenario, corner loading produces the highest stresses at the free corner, interior loading generates peak stresses at the patch base, and edge loading concentrates stress at the longitudinal interface, all consistent with classical pavement theory [34,35]. Full experimental or analytical validation against measured deflection data is acknowledged as a limitation and recommended for future work. In the absence of direct pull-off measurements for plain UPR-based PC on compacted asphalt substrates, the value of 0.78 MPa reported for a UPR-based composite bonded to asphalt pavement cores is adopted as a conservative lower-bound estimate of PC-HMA interfacial bond strength [36]. Although the formulation incorporates rubber and nanoclay modifiers absent from the plain PC mixture investigated herein, this compositional difference does not invalidate the conservative nature of the adopted value: modifier additions in UPR-based composites generally alter rather than enhance neat resin-aggregate adhesion, and the absence of such performance-enhancing treatments in the present mixture supports the adoption of 0.78 MPa as a lower-bound estimate pending direct experimental characterization [36]. Safety factor thresholds follow EN 1504-3 and ACI 548.1R: SF ≥ 2.0 for bulk tensile cracking and SF ≥ 1.0 for the interfacial debonding limit state [2,37]. A design SF of 1.5–2.0 is recommended in practice; all HIGH RISK scenarios (SF = 1.0–1.5) require mandatory surface preparation and primer application.

The finite element models were discretized using a global element size of 15 mm. To enable physically meaningful stress extraction in the vicinity of the patch-pavement interface, a local mesh refinement of 1 mm was applied specifically to the lateral faces of the repair patch and the underlying pavement interface. This local refinement was a prerequisite for the path-based stress extraction methodology: with a local element size of 1 mm, a stress extraction point at 2 mm from the singularity node is positioned at a distance equivalent to two local element lengths, ensuring the extracted value is fully mesh-resolved and free from singularity contamination. Mesh convergence was verified using Total Deformation as the control parameter, with a maximum allowable change of 1% adopted as the convergence criterion. Stress-based convergence was not used due to oscillatory behavior at geometric corners, consistent with known stress singularity effects in linear-elastic FEA [38,39]. For each analysis case, stress was extracted using a path-based linear extraction method. The location of maximum principal stress was first identified within the patch domain using ANSYS probe tools. A linear path of 15 mm length was then constructed from this point toward the patch center, and the stress value was extracted at 2 mm from the maximum stress location, corresponding to two local element lengths, thereby guaranteeing extraction outside the singularity zone. This distance was selected based on two complementary criteria: (i) it equals two local element lengths (2 × 1 mm), satisfying the mesh-size-dependent minimum distance required for singularity-free stress extraction in linear-elastic FEA [38]; and (ii) it falls within the region where Saint-Venant’s principle ensures that the stress field reflects the physically representative interfacial demand rather than a mesh-artefact peak. The bulk material strength properties and interfacial bond strengths used in the safety factor calculations are summarized in Table 7.

The interfacial bond strength values for the different material combinations are listed in Table 8, and the safety factor threshold criteria adopted from EN 1504-3 [17] and ACI 548.1R [11] are presented in Table 9.

Boundary conditions consist of fully fixed displacement constraints at the bottom of the subgrade (*U*x = *U*y = *U*z = 0) and roller supports on the four lateral vertical faces of the model (normal displacement constrained, tangential displacements free), reproducing the semi-infinite extent of the surrounding pavement. The model lateral dimensions (3.5 m × 4.5 m for JPCP and equivalent for HMA) are sufficiently large relative to the loaded area to ensure that the boundary conditions do not influence the stress field within the patch region. Each analysis case comprised approximately 275,000 ten-node tetrahedral elements and 450,000 nodes following local refinement at the patch interface.

In addition to consistency with previously published pavement-repair FEA studies [25,33] and conformity with classical pavement theory [34,35], the FEA predictions have been cross-checked against four independent literature validation benchmarks summarized in Table 10. The four benchmarks comprise: (I) an FWD-validated 3D FEA benchmark for HMA partial-depth repair; (II) two independent 3D FEA benchmarks for JPCP partial-depth repair, validated, respectively, against Westergaard’s analytical solution and the AASHO-Road-Test-validated ISLAB2000 program; (III) independent field and laboratory measured pull-off bond strength data supporting the adopted PC-JPCP bond threshold, including 18-year service performance records of polyester polymer concrete bridge-deck overlays; and (IV) independently characterized polymer-concrete material properties from compositionally analogous mixtures. Direct field validation for the specific PC patch geometries investigated remains outstanding and is identified as a priority for future work.

The first benchmark addresses flexible (HMA) pavement FEA. Chen, Wang, and Xie [25] developed an FWD-validated 3D ABAQUS model for partial-depth repair of asphalt pavements (3.9% average deviation against field measurements), reporting 0.5–1.0 MPa tensile stress in patches with *E* ≈ 18–26 GPa under HS-equivalent dual-tire loading. Our FEA predicts MPS_PC = 0.60–0.85 MPa for analogous Series-500 and Series-1000 HMA cases (Table 15 and Table 16), agreement within 15–30%. Their independent finding that PDR patch stress increases with patch depth is also consistent with our observation that MPS_PC decreases with increasing planar dimension at fixed depth.

The rigid (JPCP) pavement predictions are cross validated through two independent FEA studies, providing double redundancy. Lee, Huang, and Lin [44] developed a 3D ANSYS model for partial-depth corner repair of JPCP under HS-equivalent dual-wheel loading; geometrically and load-wise identical to our setup; validated against Westergaard’s analytical solution within 5–15%. Sharifi et al. [45] developed a 3D ABAQUS model for partial-depth repair on JPCP, cross-validated against the AASHO-Road-Test-validated ISLAB2000 program (5–10% deflection agreement), reporting ~0.5 MPa interface tensile stress for material-compatible repair. Our FEA predicts MPS_PC = 0.097–0.110 MPa for analogous JPCP scenarios; order-of-magnitude correspondence, with the difference attributable to deeper repair (76 vs. 50 mm) and corner-of-slab patch position in Sharifi et al. [45]. The agreement with two independent rigid-pavement benchmarks constitutes a double-redundant validation of our JPCP predictions.

The third line of evidence concerns the threshold value adopted for PC-JPCP interfacial bond capacity (*f*_bond,JPCP = 2.82 MPa, after [37]). Two independent measurements support this choice. WSDOT [46] conducted a comprehensive field trial of polyester polymer concrete overlays on I-90, measuring an interface bond strength of 2.89 MPa (range 2.55–3.32 MPa) per ACI 503R-93, with substrate-side failure as the predominant mode within 2.5% of the adopted threshold. Independent corroboration is provided by Wang et al. [47], who reported a mean bond strength of 2.73 MPa (ASTM C1583) for epoxy polymer concrete on PCC, again with predominantly substrate-side failure (within 3% of the adopted threshold). The 18-year service performance of 23 PPC bridge-deck overlays documented by WSDOT; with 17 of 22 inspected decks rated ‘very good to excellent’ after 5–18 years of HS-equivalent traffic; offers further empirical corroboration that polyester polymer concrete partial-depth repairs perform reliably on cementitious substrates at this stress level.

A fourth and final line of validation concerns the material parameters themselves. Abbasnejadfard et al. [49] characterized orthophthalic UPR concrete (the same resin family as in this research) reporting *E* ≈ 21,000 MPa, agreement with our value (21,247 MPa) within 1%. Their reported UPPC ultimate tensile strain of 492 × 10^−6^ establishes a strain ceiling well above the maximum strains predicted by our FEA (28–78 × 10^−6^), confirming all 36 simulated cases stay within the linear-elastic regime. Sun et al. [50] modeled a polymer-cement composite anchorage patch under HS-equivalent loading using a cohesive-zone calibrated 3D ABAQUS framework, reporting 0.46–0.85 MPa maximum principal tensile stresses; our FEA predicts 0.81–0.85 MPa for analogous 1000-D and 500-C HMA cases, agreement within 5%.

Taken together, the four independent benchmarks form a multi-benchmark indirect validation framework that compensates, within the methodological limits of a numerical study, for the absence of a direct field measurement campaign on the specific PC patch geometries investigated.

#### 2.6.3. Tire Contact Area and Loading Protocol

**Figure 2:**
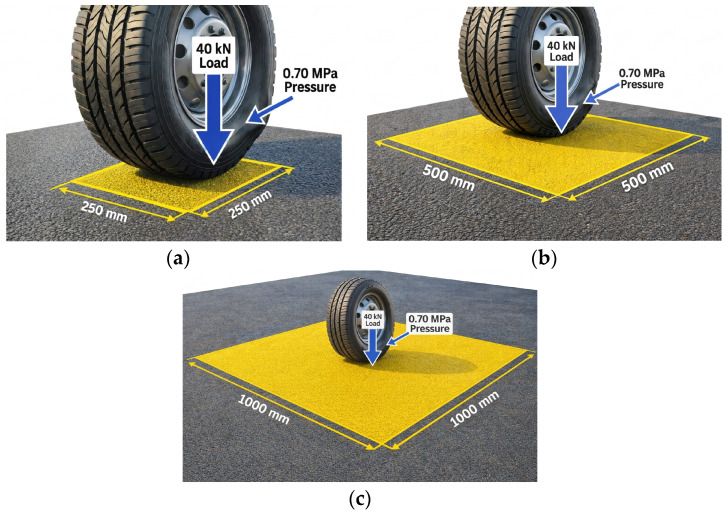
Tire contact footprint (230 × 250 mm) and the three patch geometries considered in the parametric study with the applied wheel load of 40 kN: (**a**) 250 × 250 mm patch, (**b**) 500 × 500 mm patch, and (**c**) 1000 × 1000 mm patch.

Traffic load was applied as a static uniform pressure over a rectangular footprint, with parameters drawn from AASHTO LRFD Bridge Design Specifications [51] and the contact mechanics formulations [35]. A vertical wheel load (*P*) of 40 kN was applied, representing the standard half-axle load (single wheel end) of an HS-20 design truck [35]. Regarding the contact pressure (*p*), a uniform value of 0.70 MPa (100 psi) was adopted. This value reflects modern radial tire inflation pressures, which have increased significantly from the traditional 0.55 MPa (80 psi) assumption, as reported in recent field surveys by [52]. The resulting tire contact footprint and the three patch geometries considered in the parametric study are illustrated in Figure 2.

To determine the precise contact geometry consistent with these parameters, a hybrid calculation approach was utilized. First, the required contact area (*A*c) was calculated based on the load-pressure relationship:

*A*c = *P/p* = 40,000 N/0.70 N/mm^2^ ≈ 57,143 mm^2^, (1)

Subsequently, the geometry of this area was defined to be consistent with regulatory standards. Adhering to the effective tire contact length (*L*) defined in Section 3.6.1.2.5 of the AASHTO LRFD [51], the length was fixed at 250 mm. The corresponding contact width (*W*) was then derived to satisfy the calculated area:

*W* = *A*c/*L* = 57,143 mm^2^/250 mm ≈ 228.6 mm, (2)

For the FEA modeling, these dimensions were rounded to a rectangular footprint of 230 mm (width) × 250 mm (length).

A critical aspect of this configuration was the selection of a single-tire footprint rather than the standard dual-tire configuration (510 mm width). This configuration was deliberately chosen to eliminate “bridging effects” where a wider dual-tire assembly would span across the smaller patch geometry, transferring loads to the adjacent existing pavement and artificially reducing the stress on the repair material. The single-tire model concentrates the full axle load on a smaller area, producing the most demanding localized stress state.

#### 2.6.4. Parametric Study: Geometric Sensitivity and Failure Mechanisms

The numerical investigation is structured as a multi-variable parametric study comprising 36 simulation cases that vary three parameters independently: host pavement type (flexible HMA and rigid JPCP), planar repair geometry (250 × 250, 500 × 500, and 1000 × 1000 mm), and wheel-load position (Scenarios A–D). For each pavement type, nine PC patch cases and nine corresponding traditional patch reference cases are analyzed. A subsequent dynamic amplification analysis at DIF = 1.30 (Tables 18 and 19) and DIF = 2.00 (Table 20) extends the matrix to dynamic loading conditions.

Three patch sizes were analyzed to capture the range of failure modes encountered in practice, from punching shear in small repairs to flexural cracking in large-area rehabilitation. To isolate the effect of patch footprint (length × width) from any depth-related stiffness effects, the patch thickness was held constant at 50 mm across all three geometries. This choice mirrors standard partial-depth repair practice, where patch depth follows the depth of the existing surface distress rather than the patch planar size.

Unlike joint spall repairs located at slab boundaries, this study focuses on “Mid-Slab Pothole Repairs”. For this reason, the repair patch was positioned centrally within the pavement slab to isolate the material compatibility stress from the structural edge effects of the pavement slab. Within this configuration, the critical edge loading scenarios specifically target the patch-pavement interface, where differential stiffness between the Polymer Concrete and the substrate creates the highest risk of debonding.

The loading positions applied to these geometries were selected based on the critical stress locations defined in [34] classical theory and fundamental pavement analysis principles described by [35]. To thoroughly capture the complex stress states, four primary loading scenarios were defined:

Scenario A (Corner Loading): The wheel is positioned at the transverse approach corner of the patch. As characterized by [34,35], this represents a worst-case condition where the lack of structural continuity in two directions creates a cantilever effect, leading to maximum deflection.

Scenario B (Edge Loading): The wheel is positioned over the longitudinal interface (straddling 50% on the patch and 50% on the existing pavement) to simulate the effects of lateral traffic wander, serving as the primary evaluation of interface shear strength against debonding.

Scenario C (Interior Loading): The load is centered directly on the patch to evaluate fundamental flexural strength and resistance to punching shear.

**Figure 3:**
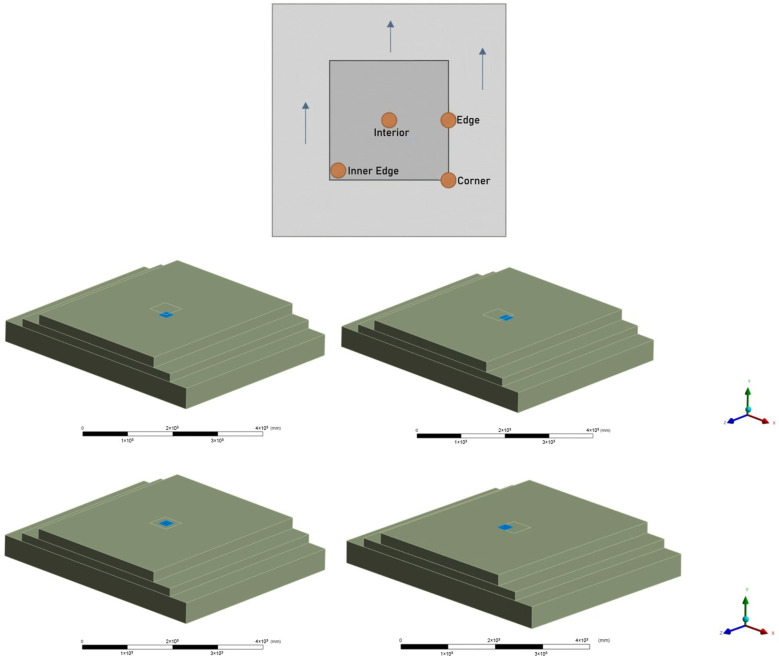
Schematic of the four loading scenarios (Corner, Edge, Interior, and Inner Edge) used in the parametric study. The plan view (top) shows the loading positions on the repair patch (darker inner square); arrows indicate the direction of traffic. The four 3D views below show the corresponding pavement models with the tire contact footprint (blue square).

Scenario D (Inner Edge Loading): The wheel footprint is placed adjacent to the longitudinal interface but remains entirely within the boundaries of the repair patch. This isolates the internal stress concentrations near the boundary without the direct load-sharing mechanism present in Scenario B. A schematic representation of the four loading scenarios is shown in Figure 3.

Based on these configurations, the simulation matrix comprises a total of nine analysis cases distributed across the three patch geometries:

Small-Scale Repair (250 × 250 mm): This geometry represents localized pothole repair. Because the single-tire footprint (230 × 250 mm) covers approximately 92% of the patch surface, edge and corner distinctions become geometrically negligible. Consequently, this size was evaluated exclusively under Scenario C (Interior Loading) (1 case). This configuration subjects the repair material to high confinement, primarily testing its resistance to punching shear and vertical compressive deformation rather than flexural bending.

Medium-Scale Repair (500 × 500 mm): Representing a standard partial-depth patch, this geometry allows the wheel load to induce significant differential deflection. To capture the full spectrum of failure mechanisms, it was analyzed under all four loading positions: Scenarios A, B, C, and D (4 cases).

Large-Scale Repair (1000 × 1000 mm): Modeled as extensive surface rehabilitation, this geometry exhibits distinct plate-like behavior due to its increased span. Similar to the medium patch, it was evaluated under all four loading positions: Scenarios A, B, C, and D (4 cases). However, the failure mechanism here shifts significantly. Under Scenario C, for instance, high flexural bending moments are induced, generating maximum tensile stresses at the bottom of the patch. This serves as the primary evaluation of the material’s tensile capacity and resistance to bottom-up cracking.

## 3. Results

Specimens were tested at 1, 3, 7, and 28 days, intervals chosen to match the decision windows commonly used in work-zone scheduling and traffic reopening protocols. These intervals are consistent with the early-age performance benchmarks referenced in work-zone mobility guidelines, rehabilitation standards, and EN structural repair specifications [53,54,55].

### 3.1. Compressive Strength Development

Compressive strength results confirmed the rapid load-carrying capability of PC. The experimentally measured values indicate that the average 1-day strength of 45.76 MPa already exceeds the early-opening thresholds commonly cited in FHWA Rapid Highway Renewal, where a minimum of 20–25 MPa is required before controlled traffic can be restored [12]. By the third day, strength increased to 85.97 MPa, indicating that more than 80% of the material’s long-term capacity had been achieved. This performance exceeds the design strength ranges recommended for heavy truck traffic corridors in AASHTO LRFD Pavement Design, making PC suitable for interstate highways, freight-dominated haul routes, and major arterial networks.

**Figure 4:**
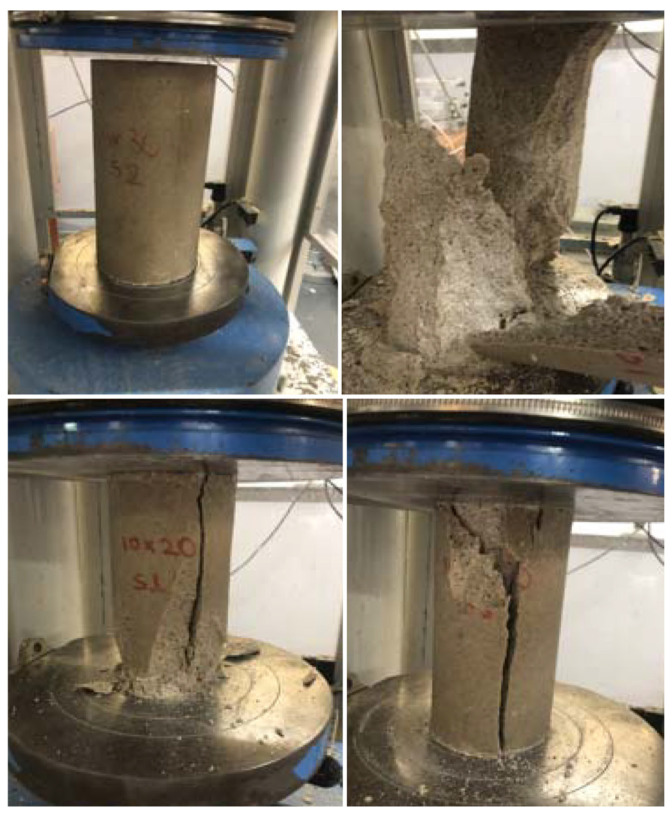
Compressive Strength Test.

Between days 7 and 28, gains were modest, reaching 93.26 MPa, confirming that polymerization is essentially complete within the first week. This behavior stands in contrast to Portland cement-based repair materials, which may require 14–28 days of hydration before reaching comparable strength. For agencies working within tight closure windows, this is a meaningful difference: it can mean the distinction between a one-day closure and a two-week lane restriction. The compressive strength testing setup is shown in Figure 4, and the results are presented in Table 11.

### 3.2. Splitting Tensile Strength Development

**Figure 5:**
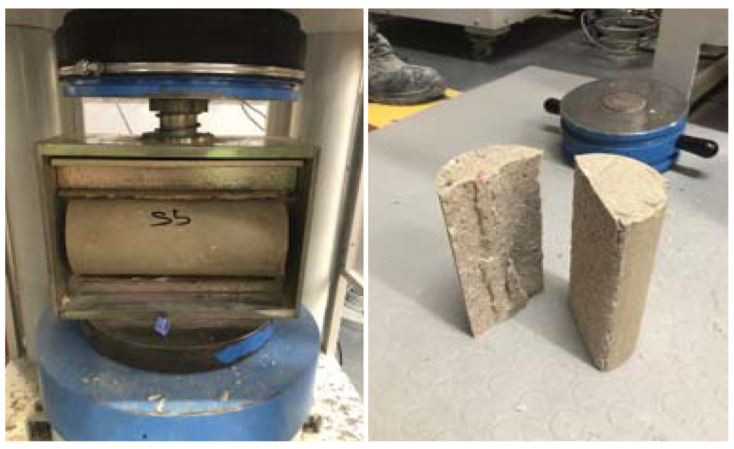
Splitting Tensile Strength Test.

Patches are routinely subjected to tensile stresses induced by braking forces, thermal gradients, and load transfer across joints. Tensile strength therefore says a great deal about how long a repair will hold in the field. Using the testing setup illustrated in Figure 5, the PC exhibited a splitting tensile strength of 5.54 MPa at 1 day (Table 12), already surpassing the bulk tensile capacity of conventional HMA patch material (1.38 MPa, Table 7) by a factor of four, confirming early-age resistance to crack initiation under traffic-induced tensile stresses [56]. By day 3, the average tensile strength increased to 7.63 MPa, a value subsequently adopted as the material capacity input for the finite element analysis, representing the early traffic reopening scenario, and continued to rise modestly, reaching 8.34 MPa at 28 days.

This strength progression suggests sufficient resistance against reflective cracking, patch debonding, and fatigue deterioration under repeated axle loading [11,36]. The stable tensile-to-compressive strength ratio (8–12%) is consistent with well-crosslinked polymer concrete systems and reflects effective stress transfer within the polymer matrix and strong aggregate–binder interfacial bonding [15,57].

### 3.3. Density

Density measurements provide an indicator of the volumetric stability and structural compatibility of polymer concrete with adjacent pavement layers. The measured density values for all specimens at each curing age are summarized in Table 13.

### 3.4. Apparent Porosity

Apparent porosity averaged 0.15% across all specimens and curing ages (Table 14), well below the threshold set for structural repair products in EN 1504-3. Recent studies emphasize that the macroscopic mechanical performance and long-term durability of polymer-based composites are primarily governed by their internal porosity and void distribution [58]. As with similar systems, such low pore connectivity is expected to limit chloride diffusion, moisture penetration, freeze–thaw deterioration, and chemical degradation, all major contributors to premature patch failure, though direct durability testing was not performed in this study.

Practically, this points toward fewer retreatments and longer patch life, a meaningful advantage in high-volume corridors where repeated closures are costly.

### 3.5. Statistical Evaluation of Experimental Results

Compressive strength (*n* = 3 per age): SD ranged from 0.75 to 2.44 MPa with CV = 0.8–5.3%; higher variability at 1 day reflects sensitivity of early-age polymerization to ambient temperature fluctuations, stabilizing to CV < 1.5% from day 3 onward. Splitting tensile strength: SD = 0.13–0.22 MPa, CV = 1.6–2.8% across all ages. Density: SD = 0.006 g/cm^3^, CV ≤ 0.3%, negligible variability confirming volumetric uniformity. Apparent porosity: SD = 0.006–0.010%, CV = 4.3–7.1%; the relatively higher CV reflects the sensitivity of the ASTM C642 measurement to specimen surface condition, though absolute variability remains below 0.01% in all cases. Outliers resulting from casting flaws or testing anomalies were excluded only when technically justified.

### 3.6. Numerical Results

To evaluate the structural integrity and mechanical scalability of the PC repair material, a thorough three-dimensional (3D) Finite Element Analysis (FEA) was conducted. The simulation framework was designed to assess the material’s response within both rigid (concrete) and flexible (asphalt) pavement structures under varying geometric constraints and critical traffic loading configurations. The present FEA study is limited to static mechanical loading conditions. Thermal effects, including differential thermal expansion between the PC patch and the host pavement arising from their distinct coefficients of thermal expansion, were not incorporated into the model. While thermal stresses may be significant under field conditions, particularly for large-area patches (1000 × 1000 mm) subjected to wide diurnal or seasonal temperature ranges, their quantification is considered beyond the scope of this investigation and is identified as a priority area for future research.

#### Safety Factor Assessment

Table 15 presents the complete safety factor assessment for HMA pavement configurations. Maximum principal stress values for the PC patch on HMA pavement range from 0.60 MPa (500-A) to 1.65 MPa (250-C), yielding bulk tensile safety factors between 4.62 and 12.70 against the 3-day tensile capacity of 7.63 MPa, all well above the SF = 2.0 minimum threshold, confirming LOW or negligible risk of internal tensile cracking across all scenarios (minimum SF = 4.62, well above the SF = 2.0 threshold). Equivalent stress safety factors range from 24.1 to 47.2 against the 3-day compressive capacity of 85.97 MPa, validating the linear-elastic FEA assumption and confirming that PC operates entirely within the elastic regime. Adopting 0.78 MPa as the conservative PC–HMA interfacial bond strength, safety factors range from 0.47 to 1.30, indicating that PC patch on HMA pavement requires careful surface preparation and primer application across all scenarios. Five out of nine HMA scenarios exhibit DEBONDING risk, 250-C (SF = 0.47), 500-C (SF = 0.92), 500-D (SF = 0.99), 1000-C (SF = 0.87), and 1000-D (SF = 0.97), while the remaining four scenarios (500-A, 500-B, 1000-A, 1000-B) show HIGH interfacial risk (SF = 1.11–1.30). These results consistently reinforce that primer application and mechanical scarification are indispensable for all PC repairs on flexible asphalt pavements. It is further noted that unsaturated polyester systems typically undergo 5–8% volumetric shrinkage during polymerization, generating residual tensile stresses at the patch-substrate interface that are not captured in the present static FEA framework. These shrinkage-induced stresses act additively with traffic-induced interfacial stresses, potentially elevating debonding risk above the values reported herein, particularly for the 250 × 250 mm patch, which already exhibits DEBONDING risk under both bond strength assumptions. This interaction represents an important limitation of the current analysis and should be addressed in future coupled mechanical-shrinkage FEA studies.

On rigid pavement, the 250 mm concrete slab absorbs a substantial share of the applied load before it reaches the patch. As a result, stress levels in the PC patch on JPCP were noticeably lower than on the HMA cases. Maximum principal stress ranges from 0.097 MPa (250-C) to 0.127 MPa (1000-B), yielding bulk tensile safety factors between 60.0 and 84.4. Interface safety factors range from 22.2 to 31.2, all indicating LOW risk of debonding when PC is applied to a properly prepared concrete substrate. The high interfacial safety margin reflects the superior bond strength of PC on Portland cement concrete (2.82 MPa, [37]) compared to asphalt (0.78 MPa, [36]), making PC particularly well-suited for rigid pavement repair.

**Table 16:** Safety Factor Assessment—JPCP (Rigid) Pavement (PC Patch, Patch-Only Region).

Scenario	Traditional Patch Max PS (MPa)	PC Patch Max PS (MPa)	PC SF Tensile	Tensile Risk	PC SF Interface	Interface Risk	PC SF Compress.
250-C	0.0565	0.0978	78.05	LOW	28.85	LOW	143.2
500-A	0.0830	0.1007	75.74	LOW	27.99	LOW	72.1
500-B	0.1298	0.1272	60.01	LOW	22.18	LOW	100.2
500-C	0.1172	0.1135	67.20	LOW	24.84	LOW	185.0
500-D	0.0835	0.0904	84.37	LOW	31.18	LOW	72.6
1000-A	0.1179	0.1103	69.20	LOW	25.58	LOW	178.5
1000-B	0.1286	0.1260	60.58	LOW	22.39	LOW	160.5
1000-C	0.1195	0.1116	68.36	LOW	25.26	LOW	184.4
1000-D	0.1174	0.1072	71.21	LOW	26.32	LOW	100.0

Note: PC bulk tensile capacity = 7.63 MPa (3-day experimental); PC-JPCP bond = 2.82 MPa [37]. All SF values for PC on JPCP indicate LOW risk across all scenarios. Traditional patch Max PS values for all JPCP scenarios fall within the consistent range of 0.056–0.130 MPa, confirming uniform stress distribution across patch sizes and loading positions. The Concrete_Patch (traditional) and PC_Patch show comparable stress levels on rigid pavement, reflecting the dominant load distribution capacity of the 250 mm JPCP slab.

**Figure 6:**
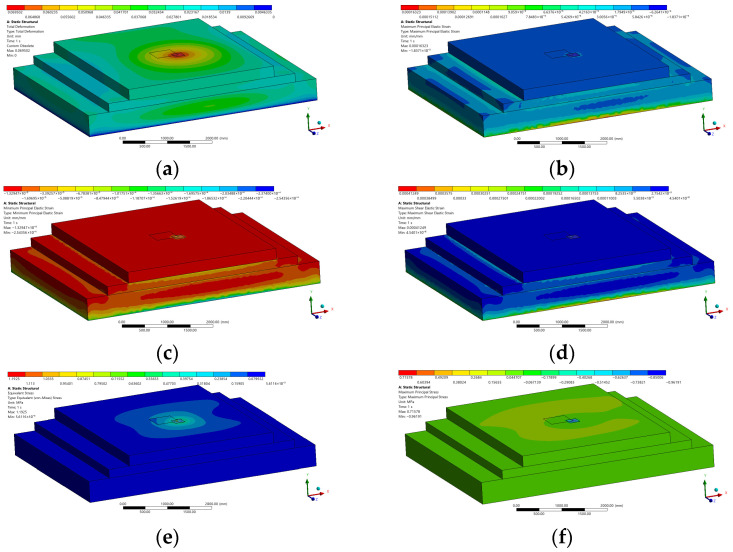
ANSYS FEA results for JPCP pavement with PC patch under 500 × 500 mm Corner Loading (Scenario A): (**a**) total deformation, (**b**) maximum principal elastic strain, (**c**) maximum value of minimum principal elastic strain, (**d**) maximum shear elastic strain, (**e**) equivalent (Von Mises) stress, (**f**) maximum principal stress distribution across the full pavement model.

**Figure 7:**
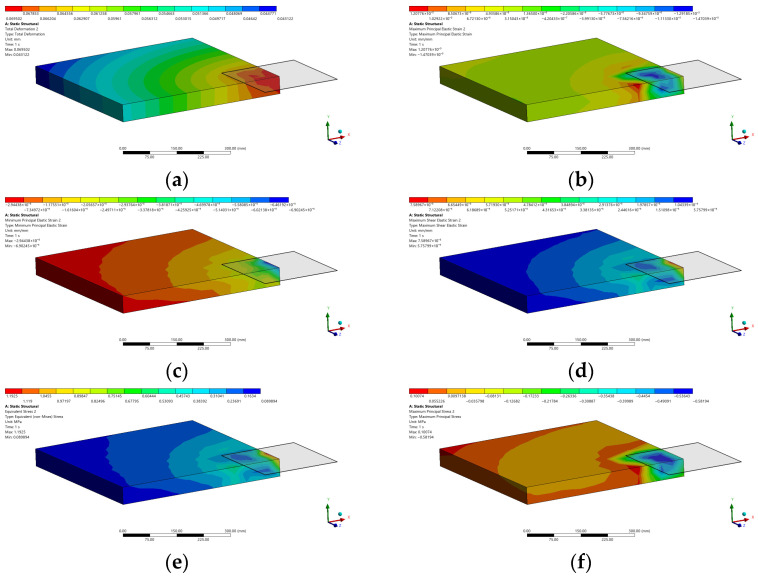
ANSYS FEA results for PC patch under 500 × 500 mm Corner Loading (Scenario A): (**a**) total deformation, (**b**) maximum principal elastic strain, (**c**) maximum value of minimum principal elastic strain, (**d**) maximum shear elastic strain, (**e**) equivalent (Von Mises) stress, (**f**) maximum principal stress.

Representative ANSYS contour plots for the JPCP + PC configuration under 500 × 500 mm Corner Loading (Scenario A) are presented in Figure 6 and Figure 7, illustrating the total deformation, principal strains, shear strain, and stress distributions across the full pavement model and the PC patch region, respectively.

Table 17 presents the total system deformation for all nine analysis cases. PC reduces maximum system deformation by 4.3–15.3% compared to traditional HMA patches on flexible pavements, with the greatest benefit observed at large patch sizes under interior loading. On JPCP, differences remain structurally negligible (<0.5%), consistent with the dominant load distribution role of the 250 mm concrete slab.

To assess the effect of dynamic loading, a Dynamic Impact Factor of 1.30 was applied to all extracted stresses. Table 18 and Table 19 present the complete static versus dynamic safety factor comparison for HMA and JPCP configurations, respectively. An additional sensitivity analysis at DIF = 2.00 is reported in Table 20, capturing the 95th-percentile spatial-repeatability envelope of dynamic axle forces after Goenaga et al. [59]; together, the DIF = 1.30 results in Table 18 and Table 19 and the DIF = 2.00 results in Table 20 bracket the realistic dynamic range supported by recent studies [60,61,62,63].

At DIF = 2.00, every one of the nine HMA scenarios falls into the DEBONDING classification (interfacial SF = 0.23–0.65), whereas the JPCP scenarios all remain in the LOW classification, with the lowest interfacial SF being 11.09. The mechanistic interpretation and design implications of these findings are addressed in Section 4.4.

## 4. Discussion

### 4.1. Mechanical Performance and Microstructural Interpretation

The near-zero porosity recorded across all curing ages traces directly back to how unsaturated polyester resin hardens. During the MEKP/CoNap-initiated radical polymerization, the liquid resin undergoes copolymerization and crosslinking with styrene monomer, transforming from a fusible, soluble liquid into an insoluble, infusible three-dimensional network structure that uniformly envelops aggregate surfaces [64]. Unlike in Portland cement concrete, where a water-rich Interfacial Transition Zone (ITZ) forms around aggregate particles due to water migration and localized calcium hydroxide accumulation, creating a structurally weaker region prone to microcracking, the polymer binder in PC undergoes a waterless polymerization reaction that eliminates the conditions responsible for ITZ weakness. The crosslinked polymer film adheres to aggregate surfaces through both mechanical interlocking with surface irregularities and physico-chemical adhesion, producing a uniform, low-void matrix-aggregate interface [7,65]. The quality and completeness of this crosslinking reaction is the primary determinant of PC’s macroscopic performance: well-crosslinked mixtures exhibit sealed interstitial pores, high chemical resistance, and strong aggregate–binder bonding, all of which are reflected in the consistently low porosity (0.15%) and high compressive and tensile strengths reported in the present study [1,3].

**Figure 8:**
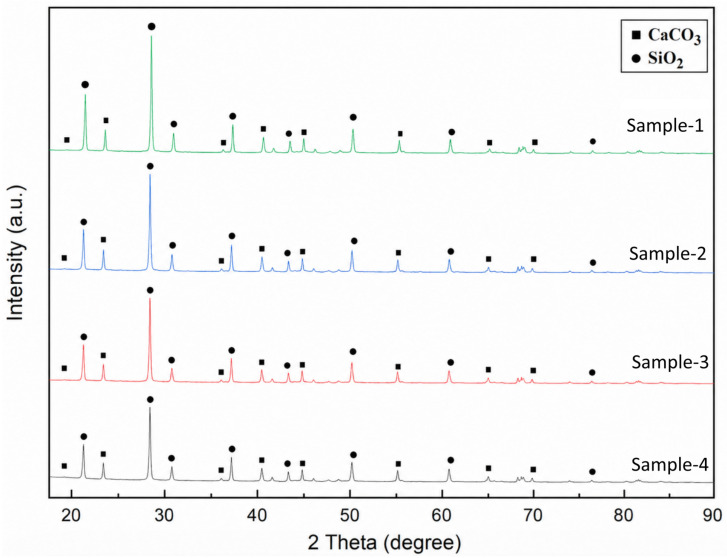
XRD images obtained from the specimens.

The X-ray diffraction (XRD) patterns obtained from samples are presented in Figure 8. The diffraction analyses were carried out to investigate the crystalline structure, phase composition, and microstructural characteristics of the produced materials. According to the diffraction peaks observed within the 2*θ* range of 20–90°, the dominant crystalline phases detected in all specimens were identified as silicon dioxide (SiO_2_, quartz) and calcium carbonate (CaCO_3_), reflecting the mineralogical constituents of the natural river-bed aggregate. The characteristic peaks corresponding to these phases were marked on the diffraction patterns using square and circular symbols, respectively.

A strong and sharp diffraction peak observed around 2*θ* ≈ 26.6° corresponds to the crystalline SiO_2_ phase, indicating the presence of quartz-rich mineral content in the microstructure. The relatively high intensity and narrow width of this peak suggest a well-developed crystalline structure and comparatively larger crystallite domains. In addition, several secondary SiO_2_-related reflections detected between approximately 36° and 68° further confirm the persistence of silica-based crystalline phases throughout all investigated specimens.

The CaCO_3_ reflections were identified near 29.4°, 39–48°, and approximately 57–69°. These reflections are consistent with the calcium content measured by EDS (Ca: 2.9 wt.%) and indicate the presence of calcite grains within the natural river-bed aggregate. The simultaneous presence of SiO_2_ and CaCO_3_ phases demonstrates that the aggregate possesses a heterogeneous mineralogical composition.

Furthermore, the relatively narrow diffraction peaks observed in several regions imply low amorphous content and good crystalline ordering within the material structure. Minor peak broadening detected in some reflections may be associated with microstrain development and reduced crystallite size. Therefore, the Williamson-Hall (W-H) approach was employed to evaluate the crystallite size and lattice strain contributions simultaneously. According to the Williamson–Hall method, the relationship between peak broadening and diffraction angle can be expressed as:

(3) $$\beta \cos\theta =\frac{k\lambda }{D}+4\varepsilon \sin\theta$$

where *β* represents the full width at half maximum (FWHM), *θ* denotes the Bragg diffraction angle, *λ* is the X-ray wavelength, *k* is the shape factor, *D* is the crystallite size, and *ε* represents the lattice strain. The linear relationship between *β*cos *θ* and 4sin *θ* enables simultaneous estimation of crystallite size from the intercept and lattice strain from the slope of the fitted line.

Overall, the XRD results indicate that the investigated specimens possess a stable crystalline microstructure composed of quartz and calcite phases. These crystalline constituents originate from the natural river-bed aggregate and are preserved within the cured polymer matrix.

Scanning Electron Microscopy (SEM) analyses were conducted using a field-emission scanning electron microscope to investigate the surface morphology, fracture characteristics, and microstructural features of the specimens. Prior to imaging, all samples were sputter-coated with a thin layer of gold in order to improve electrical conductivity and enhance image quality during SEM examination.

SEM micrographs revealed that the specimens exhibited a dense and irregular particle morphology with relatively limited visible voids and microcracks. The matrix structure appeared compact and continuous, indicating effective bonding between the matrix and aggregate phases. In particular, the matrix-aggregate interface demonstrated strong adhesion characteristics, suggesting efficient stress transfer throughout the material structure. Compared to conventional cementitious materials, the absence of extensive capillary pore networks was clearly evident, supporting the low porosity (0.15%) measured experimentally in Section 3.4.

**Figure 9:**
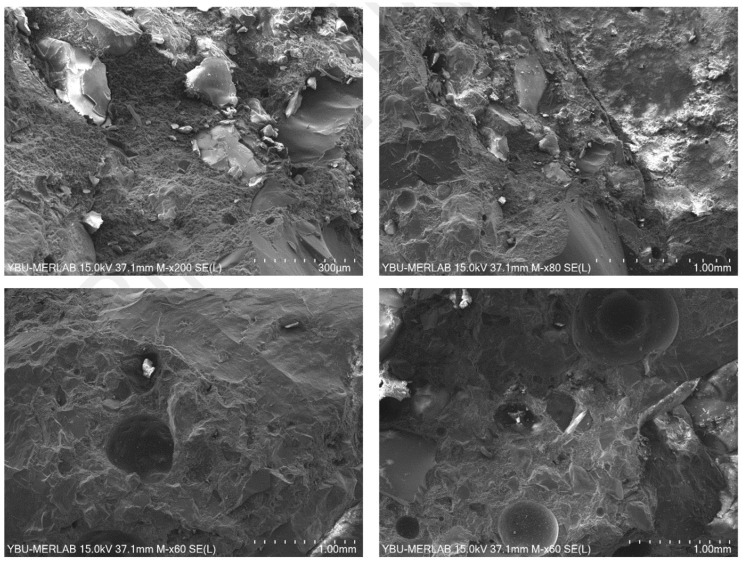
SEM images obtained from the specimens.

The SEM images obtained at different magnification levels showed tightly packed particles embedded within the matrix together with locally distributed isolated pores (Figure 9). Although several spherical voids and discontinuities were identified in some regions, these defects appeared limited and non-uniformly distributed. The absence of interconnected pore channels indicates that the material possesses a relatively dense internal structure with acceptable microstructural integrity. In addition, the relatively smooth and compact surface texture observed in the micrographs is consistent with the high compressive (85.97 MPa) and splitting tensile (7.63 MPa) strength values obtained experimentally at 3 days.

The fracture surfaces exhibited rough and irregular crack propagation paths rather than straight brittle fracture planes. Such tortuous crack trajectories indicate that the microstructure contributed to energy dissipation during crack development. In several regions, aggregate particles remained strongly attached to the surrounding matrix, demonstrating effective resin-aggregate bonding. The resin matrix (RM) and aggregate particles (AG) could be clearly distinguished in the micrographs. The dense morphology and limited occurrence of wide pores indicate effective penetration of the polymer binder into aggregate surface irregularities and pore spaces, thereby improving interfacial bonding performance.

Furthermore, some smooth and glassy regions observed on the fracture surfaces may correspond to polymer-rich phases or locally densified binder accumulations. These compact regions likely contributed to reduced permeability and enhanced mechanical stability. The coexistence of dense matrix zones and mineral particles is also consistent with the XRD results, which confirmed the presence of silica and carbonate-based crystalline phases within the material composition.

Overall, the SEM observations demonstrated that the investigated specimens possessed a compact and well-integrated microstructure characterized by strong matrix-aggregate bonding, limited microcracking, and relatively low pore connectivity. The dense internal morphology and improved interfacial characteristics are considered major factors contributing to the superior compressive strength, tensile performance, and low porosity of the specimens.

**Figure 10:**
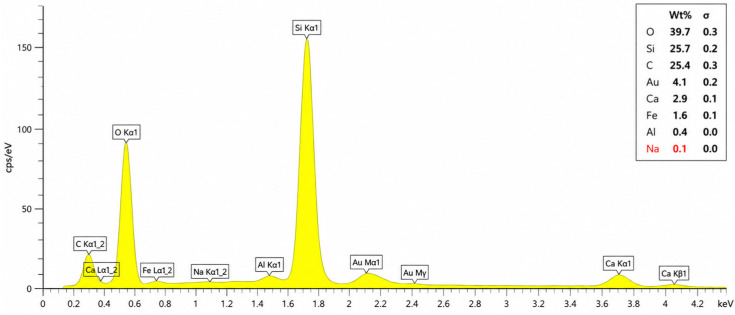
Energy-dispersive X-ray spectroscopy (EDS) spectrum and elemental composition analysis.

The elemental composition of the investigated specimen was evaluated using energy-dispersive X-ray spectroscopy (EDS) in conjunction with SEM imaging. The obtained EDS spectrum demonstrated that the material predominantly consisted of oxygen (O), silicon (Si), and carbon (C), indicating the coexistence of silica-rich and carbon-containing phases within the microstructure (Figure 10). According to the quantitative elemental analysis, oxygen was detected as the dominant element with a weight percentage of approximately 39.7 wt.%, followed by silicon (25.7 wt.%) and carbon (25.4 wt.%). The relatively high silicon content confirms the presence of silica-based mineral phases, which is fully consistent with the XRD results that identified crystalline SiO_2_ peaks.

The high oxygen concentration observed in the spectrum is associated with oxide-based compounds and silicate structures within the matrix. The considerable carbon content (25.4 wt.%) primarily reflects the cured unsaturated polyester resin matrix. The dominance of Si (25.7 wt.%) and O (39.7 wt.%) signals confirms the predominantly siliceous composition of the aggregate skeleton, consistent with the SiO_2_ peaks observed in the XRD analysis, while the calcium signal reflects the carbonate constituents of the natural river-bed aggregate.

Minor amounts of calcium (Ca), iron (Fe), aluminum (Al), and sodium (Na) were also detected in the spectrum. These signals reflect the mineralogical constituents of the natural river-bed aggregate (Section 2.1).

The gold (Au) peak observed in the spectrum originated from the thin gold coating applied prior to SEM imaging to enhance electrical conductivity and improve imaging quality. Therefore, the detected Au content is associated with sample preparation rather than the intrinsic composition of the material.

The dominance of Si and O peaks, together with the calcium and other trace elemental signals, reflects the mixed mineralogical composition of the natural river-bed aggregate. Furthermore, the relatively homogeneous elemental distribution and absence of excessive impurity peaks indicate a stable and chemically compatible matrix system. These findings support the SEM observations, which revealed a dense and compact morphology with strong matrix-aggregate bonding and limited pore connectivity.

Overall, the EDS analysis confirmed that the material mainly consisted of silica-rich and carbonate-bearing mineral phases originating from the natural river-bed aggregate, together with the cured polymer matrix. The combined SEM-EDS observations indicate that the dense microstructure and compatible elemental composition contributed significantly to the improved mechanical performance and low porosity of the specimens.

### 4.2. Transportation Engineering Implications

As presented in Table 13, density values remained remarkably consistent across all curing ages, averaging 2.11 g/cm^3^. Such volumetric stability suggests minimal shrinkage, absence of internal void formation, and uniform binder distribution. From a transportation engineering perspective, maintaining a density range comparable to asphalt and Portland cement concrete ensures structural compatibility and prevents differential settlement, an issue specifically discussed in research related to pavement distress identification and EN 13108 asphalt mix design standards [18,66,67].

This stability supports reliable load transfer behavior and surface smoothness, reducing the potential need for grinding, re-leveling, or additional finishing operations after installation.

Collectively, the results demonstrate that PC satisfies multiple operational requirements emphasized by international transportation agencies. Its rapid strength gain enables reopening of high-volume roadway segments within 24 h, supporting the short work-zone occupation times. Its tensile and compressive resistance satisfy pavement performance expectations defined by AASHTO for structural overlays and full-depth repairs. Its low porosity and stable density are consistent with potentially improved long-term durability relative to conventional repair materials, pending direct characterization through freeze-thaw cycling, chloride diffusion, and accelerated aging tests aligned with EN performance-based repair frameworks. PC presents itself as a viable, performance-driven repair material for emergency patching, utility trench reinstatement, intersection rehabilitation, toll plaza resurfacing, and localized distress remediation along motorway infrastructures. From a cost perspective, unsaturated polyester resin is substantially more expensive per unit volume than hot mix asphalt or Portland cement concrete. However, the rapid curing enabling same-day lane reopening reduces work-zone occupation costs, and the low-porosity microstructure suggests fewer repeat interventions over the pavement lifecycle. A formal life-cycle cost analysis comparing PC with conventional repair options is identified as a necessary step before widespread adoption in maintenance specifications.

### 4.3. Interface Debonding Risk and Field Practice Recommendations

Across all 36 cases, PC showed no sign of approaching its tensile capacity. On HMA pavement, safety factors ranged from 4.62 to 12.70 under static loading and from 3.56 to 9.77 when a dynamic impact factor of 1.30 was applied. On JPCP, the corresponding ranges were 60.0–84.4 and 46.1–64.9, the rigid slab simply does not generate enough bending to challenge the material. These margins are significantly higher than those of traditional patches. For comparison, traditional HMA patch tensile safety factors on HMA pavement, calculated as SF = *f*_t,HMA/MPS_traditional = 1.38 MPa/MPS (where MPS values are listed in Table 15, Traditional Patch Max PS column), range from 5.59 (1000-B) to 14.26 (250-C). While these ratios appear comparable to those of PC, the critical distinction lies in the capacity: PC’s tensile capacity (7.63 MPa) is 5.5 times that of HMA (1.38 MPa). This means that for the same applied stress, PC carries a structurally superior reserve, and the PC patch would still be intact at stress levels that would already cause internal cracking in an HMA patch. Von Mises safety factors for PC spanned 24.1 to 185.0, placing the material well within the elastic range at 3-day strength. This validates the elastic constitutive model used in the analysis.

The interfacial analysis reveals a critical distinction between PC performance on flexible and rigid pavements. Consistent with FEA-based debonding analyses of polymer-bonded composites on concrete substrates, which consistently identify the polymer-concrete interface as the governing failure plane under concentrated loading [68], the present results confirm that the PC patch-pavement interface represents the most critical structural limit state, particularly on flexible pavements. On JPCP, all PC cases demonstrate LOW interfacial risk (SF = 22.2–31.2), reflecting the high bond strength between PC and Portland cement concrete substrates (2.82 MPa, [37]). Standard surface scarification and cleaning are sufficient for PC repair on rigid pavements. Adopting 0.78 MPa as the conservative PC-HMA bond strength, five out of nine HMA scenarios exhibit DEBONDING risk (250-C, 500-C, 500-D, 1000-C, and 1000-D; SF = 0.47–0.99), while the remaining four (500-A, 500-B, 1000-A, 1000-B) show HIGH interfacial risk (SF = 1.11–1.30). The 250-C critical case arises because a single-tire footprint (230 × 250 mm) covers approximately 92% of the small patch, concentrating interfacial shear and tensile stress at the patch corner and producing the lowest safety factor (SF = 0.47). These findings reinforce that PC repair on flexible pavements must be accompanied by mechanical scarification and primer application prior to placement, regardless of patch geometry. In contrast, traditional HMA patch on HMA pavement exhibits interfacial safety factors of 2.02–5.17 (static, patch-only region; SF = *f*_0_,HMA/MPS = 0.50 MPa/MPS, ranging from SF = 2.02 at scenario 1000-B to SF = 5.17 at scenario 250-C), indicating MEDIUM to LOW interfacial risk under static loading for conventional repairs on asphalt substrates.

### 4.4. Structural Performance and Deformation

The stiffness differential between PC (*E* = 21,247 MPa) and HMA (*E* = 3500 MPa) drives a load concentration effect: the stiffer patch attracts stress from the surrounding pavement. For example, in the 500-A scenario on HMA pavement, the maximum principal stress within the PC patch (patch-only region) is 0.60 MPa, while the maximum principal stress in the full pavement model, which occurs in the surrounding HMA layer outside the patch boundary, is 0.91 MPa. This means the stiffer PC patch absorbs a disproportionately large share of the applied load, reducing the stress demand on the adjacent asphalt and thereby shielding it from load-induced damage. By drawing stress into the repair zone rather than dispersing it into the adjacent pavement, PC actively protects the surrounding HMA from fatigue-driven damage propagation. PC patch also reduces total system deformation by 4.3–15.3% compared to traditional HMA patch on flexible pavements, with the greatest benefit at larger patch sizes under interior loading. On JPCP, deformation differences are small in absolute terms, though not uniformly negligible. In scenarios where the rigid concrete slab governs system stiffness, JPCP + PC and JPCP + Concrete deformations are virtually identical (differences < 0.5%). However, in certain scenarios (e.g., 1000-B and 1000-C), JPCP + PC deformation marginally exceeds that of JPCP + Concrete by 0.001–0.002 mm. This is physically consistent: PC’s elastic modulus (21,247 MPa) is approximately 23% lower than that of the concrete patch (27,500 MPa), so under equivalent load, the thinner PC patch (50 mm) exhibits slightly greater local elastic deformation within the patch zone. Given the dominant role of the 250 mm concrete slab, these differences are structurally inconsequential and fall well within the tolerance range for surface smoothness in highway repair applications.

The elastic modulus and strength of polymer-based materials can shift with loading rate and internal microstructural condition [69]. The DIF sensitivity results yield two engineering findings. On HMA pavement, the interface check governs the response across the entire DIF range; at DIF = 2.00, every one of the nine HMA scenarios falls into the DEBONDING classification, reinforcing the central recommendation that PC repairs on flexible pavements require primer application and mechanical scarification regardless of patch geometry. On JPCP, every safety factor remains in the LOW classification at every DIF tested, with the lowest interfacial SF of 11.09 even at DIF = 2.00, confirming that PC repairs on rigid pavements are robust to dynamic loading. These results should be interpreted as a double-conservative estimate: the present analysis already employs a single-tire footprint (230 × 250 mm), which concentrates load more severely than the standard dual-tire configuration of actual HS-20 axle loading. A fully coupled vehicle-pavement dynamic analysis incorporating axle suspension dynamics, surface roughness power spectra, and tire–pavement interaction is identified as the natural next step.

## 5. Conclusions

The experimental results provide consistent evidence of PC’s early-age mechanical performance; the reported values are based on *n* = 3 specimens per age, and broader statistical confirmation with larger sample sets is recommended before specification adoption. Compressive strength reached 45.76 MPa within one day and 85.97 MPa by day three. Both values satisfy international guidelines for early traffic reopening standards. Splitting tensile strength values, which increased from 5.54 MPa at one day to 7.63 MPa at three days and reached 8.34 MPa at twenty-eight days, revealed strong resistance to crack initiation and propagation, an essential requirement for pavement patches exposed to braking, thermal gradients, and repeated axle loading. The consistently stable density and extremely low porosity (average 0.15%) are consistent with potentially superior long-term durability and reduced permeability, suggesting improved resistance to freeze-thaw cycles, chloride exposure, and moisture-driven deterioration; however, these properties require direct experimental validation through dedicated durability testing.

Across all measured parameters, PC compares favorably to conventional asphalt and cement-based repair materials, and the advantage is most pronounced precisely where it matters most: high-volume corridors where closures are costly and durability is non-negotiable. Its curing kinetics, mechanical capacity, and microstructural density are consistent with the performance benchmarks set by FHWA, AASHTO, EN, and PIARC for rapid pavement rehabilitation. As agencies shift toward performance-based maintenance contracting, PC’s profile, fast, strong, and durable, positions it well as a candidate material for rapid repair specifications.

The numerical investigation confirms that PC patch maintains a large structural reserve against internal tensile cracking on both flexible and rigid pavements, with bulk tensile safety factors ranging from 4.62 to 84.4 across all 36 analysis cases, based on 3-day experimental strength values (*f*_t = 7.63 MPa, *f*_c = 85.97 MPa) consistent with the FEA model inputs. On rigid (JPCP) pavement, PC demonstrates particularly favorable performance with interfacial safety factors exceeding 22.0, indicating that standard surface preparation is sufficient for reliable PC repair. On flexible (HMA) pavement, adopting 0.78 MPa as a conservative lower-bound estimate of PC–HMA interfacial bond strength, five scenarios exhibit DEBONDING risk (250-C, 500-C, 500-D, 1000-C, and 1000-D; SF = 0.47–0.99), while the remaining four scenarios show HIGH interfacial risk (SF = 1.11–1.30). All HMA configurations require mechanical scarification and primer application prior to PC placement. The deformation analysis reveals that PC reduces total system deformation by up to 15.3% compared to traditional HMA patch on flexible pavements, with the greatest benefit at large patch sizes under interior loading. Laboratory results confirm the potential of PC for rapid pavement repair, but several practical and methodological limitations warrant acknowledgement. Polymerization shrinkage and differential thermal expansion at the patch-substrate interface are not represented in the static linear-elastic framework adopted. Unsaturated polyester resins typically undergo 5–8% volumetric shrinkage during polymerization; although the dense aggregate skeleton and low resin content (13%) of the present mixture physically restrain volumetric changes, and viscoelastic relaxation mitigates early-age stress build-up [70], residual interfacial stresses remain additive to traffic-induced demand. The CTE mismatch between UPR-PC and PCC [71] may further induce secondary thermal stresses under diurnal cycling. An ongoing Phase II experimental study using freeze-thaw chamber testing addresses these effects directly. In addition, although the FEA predictions have been cross-checked against four independent literature validation benchmarks (Section 2.6.2), direct field validation for the specific PC patch geometries investigated remains outstanding; a controlled instrumented field programme with strain gauges, pull-off bond tests (ASTM C1583), and Falling Weight Deflectometer measurements is identified as essential for completing the validation chain prior to specification adoption. Additionally, styrene-based unsaturated polyester systems release volatile organic compounds (VOC) during mixing and curing; in enclosed or poorly ventilated field settings, this raises occupational health and environmental considerations that practitioners must address through appropriate ventilation and personal protective equipment. The present study is further limited to a single resin formulation and mixture proportion; generalization of the findings to other polyester grades, aggregate types, or resin-to-aggregate ratios should be approached with caution until confirmed by further experimental evidence. The present study did not investigate fatigue life, impact resistance, thermal expansion compatibility with asphalt and Portland cement concrete, or bond strength to existing pavement substrates, parameters that play a critical role in long-term field performance. Field trials across different climatic zones and traffic compositions would help establish realistic service life estimates and clarify how deterioration mechanisms develop under in-service conditions. Future studies may also explore mix optimization, resin chemistry modifications, recycled aggregate incorporation, and cost-benefit analysis relative to traditional repair materials. Finally, integrating pavement management system modeling and life-cycle assessment could further support decision-making for transportation authorities seeking sustainable, durable, and operationally efficient repair solutions.

## Figures and Tables

**Table 1:** Technical Properties of Unsaturated Polyester Resin.

Property	Value	Test Condition
Viscosity	~320 cP	20 °C
Gel time	~5.3 min	Ambient temperature
Acid value	21.3 mg KOH/g	Standard titration
Flash point	33 °C	Closed cup
Density	1.12 g/cm^3^	25 °C
Appearance	Clear, light-yellow liquid	—

**Table 2:** Properties of MEKP Curing Agent.

Property	Value	Test Condition
Viscosity	19 mPa·s	20 °C
Active oxygen content	~9.7%	Manufacturer data
SADT	~60 °C	Safety sheet
Flash point	>80 °C	Closed cup
Density	1.17 g/cm^3^	25 °C
Physical state	Transparent liquid	—

**Table 3:** Properties of Cobalt Naphthenate Accelerator.

Property	Value	Test Condition
Cobalt content	1.5%	Manufacturer data
Viscosity	300 mPa·s	20 °C
Density	0.92 g/cm^3^	25 °C
Appearance	Dark violet liquid	—
Function	Polymerization accelerator	—

**Table 4:** Aggregate Gradation and Physical Characteristics.

Size Fraction	Percentage by Weight (%)	Bulk Specific Gravity	Shape
0–1 mm	35	2.63	Rounded–subangular
1–3 mm	40	2.64	Angular
3–5 mm	25	2.65	Angular
Total	100	—	—

**Table 5:** Final PC Mix Proportions.

Component	Proportion by Weight (%)
Unsaturated polyester resin	13
MEKP curing agent	1.25 (of resin mass)
Cobalt accelerator	1.50 (of resin mass)
Graded aggregates	87
Total	100

**Table 6:** Material Properties and Layer Thickness Used in FEA Models [15].

Pavement Type	Layer	Thickness (mm)	Young’s Modulus E (MPa)	Poisson’s Ratio (ν)	Density (ρ)	Source
Rigid	Concrete Slab (JPCP)	250	27,500	0.20	2400 kg/m^3^	Table 11-5 of [15]
	Granular Subbase	200	275	0.35	2150 kg/m^3^	Table 11-10 of [15]
	Subgrade (Soil)	500	115	0.45	1800 kg/m^3^	Table 11-10 of [15]
Flexible	Asphalt (HMA)	150	3500	0.35	2300 kg/m^3^	Table 11-3 of [15]
	Granular Base	300	275	0.40	2150 kg/m^3^	Table 11-10 of [15]
	Subgrade (Soil)	500	115	0.45	1800 kg/m^3^	Table 11-10 of [15]
Repair	Polymer Concrete (PC)	50	21,247	0.18	2110 kg/m^3^	Experimental Data

**Table 7:** Bulk Material Strength Properties Used in Safety Factor Calculations.

Material	Bulk Tensile Strength (MPa)	Bulk Compressive Strength (MPa)	Interface Bond Strength (MPa)	Host Pavement	Source
Polymer Concrete (PC)	7.63	85.97	0.78(HMA) 2.82 (JPCP)	HMA/JPCP	This study (bulk properties); see Table 8 (bond strength)
Hot Mix Asphalt (HMA)	1.38	4.00	0.50	HMA	[40]
Portland Cement Concrete (PCC)	3.27	27.58	1.50	JPCP	[41,42]

Notes: PC values correspond to 3-day curing age (f_t = 7.63 MPa, f_c = 85.97 MPa), consistent with FEA model inputs and early traffic reopening scenario. HMA upper-bound adopted for most conservative traditional material comparison.

**Table 8:** Interfacial Bond Strength Values and References.

Interface	Bond Strength (MPa)	Reference
PC-Asphalt (HMA) (PCC substrate)	0.78	[36]
PC-Portland Cement Concrete (JPCP)	2.82	[37]
HMA-HMA (tack coat)	0.50	[43]

Notes: PC-HMA bond strength: 0.78 MPa (UPR-based composite on asphalt pavement cores; adopted as conservative estimate for risk classification).

**Table 9:** Safety Factor Threshold Criteria.

Check	SF < 1.0	SF 1.0–2.0	SF 2.0–4.0/>4.0
Bulk Tensile (Internal Cracking)	FAILED	HIGH RISK	MEDIUM/LOW RISK
Interface Bond (Debonding)	DEBONDING	HIGH RISK (Primer required)	MEDIUM/LOW RISK
Compressive/Von Mises	FAILED	HIGH	MEDIUM/LOW

Thresholds per EN 1504-3 and ACI 548.1R; SF = 1.0 is the theoretical onset of bond failure. Design SF of 1.5–2.0 is recommended in practice; HIGH RISK scenarios require mandatory primer [11,17].

**Table 10:** Indirect Validation of Present FEA Against Four Independent Validation Benchmarks.

Benchmark	Validation/Threshold Support	Key Reference	Independent Validation	Agreement
I	HMA partial-depth repair, 3D FEA	[25]	FWD field measurements	MPS_PC: 0.5–1.0 vs. 0.60–0.85 MPa; 15–30%
IIa	JPCP corner repair, 3D FEA	[44]	Westergaard analytical solution	Identical loading/geometry; methodology corroboration
IIb	JPCP corner repair, 3D FEA	[45]	ISLAB2000 (vs. AASHO Road Test)	~0.5 vs. 0.097–0.110 MPa; order of magnitude
IIIa	Adopted threshold consistency check	[46]	ACI 503R-93 on I-90	2.89 vs. 2.82 MPa; 2.5%
IIIb	Adopted threshold consistency check	[47]	ASTM C1583 [48]	2.73 vs. 2.82 MPa; 3%
IVa	UPPC E and tensile strength	[49]	Direct testing, Ø60 × 120 mm	E: 21,000 vs. 21,247 MPa; 1%
IVb	Polymer-cement composite MPS, 3D FEA	[50]	Cohesive zone calibrated	0.46–0.85 vs. 0.81–0.85 MPa; 5%

**Table 11:** Compressive Strength Results.

Curing Age	Specimen	Max Load (kN)	Strength (MPa)
1 day	PC-1	71.12	44.45
	PC-2	70.81	44.26
	PC-3	77.72	48.58
	Average	73.22	45.76
3 days	PC-4	136.12	85.08
	PC-5	139.81	87.38
	PC-6	136.72	85.45
	Average	137.55	85.97
7 days	PC-7	142.25	88.92
	PC-8	145.66	91.10
	PC-9	144.02	90.08
	Average	143.98	90.03
28 days	PC-10	147.91	92.52
	PC-11	150.34	94.01
	PC-12	149.18	93.25
	Average	149.14	93.26

SD/CV: 1-day (2.44 MPa, 5.3%); 3-day (1.24 MPa, 1.4%); 7-day (1.09 MPa, 1.2%); 28-day (0.75 MPa, 0.8%). Full discussion in Section 3.5.

**Table 12:** Splitting Tensile Strength Results.

Curing Age	Specimen	Strength (MPa)
1 day	PC-T1	5.42
	PC-T2	5.68
	PC-T3	5.51
	Average	5.54
3 days	PC-T4	7.42
	PC-T5	7.85
	PC-T6	7.63
	Average	7.63
7 days	PC-T7	7.85
	PC-T8	8.02
	PC-T9	7.64
	Average	7.84
28 days	PC-T10	8.21
	PC-T11	8.47
	PC-T12	8.33
	Average	8.34

SD/CV: 1-day (0.13 MPa, 2.4%); 3-day (0.22 MPa, 2.8%); 7-day (0.19 MPa, 2.4%); 28-day (0.13 MPa, 1.6%). Full discussion in Section 3.5.

**Table 13:** Density Measurements.

Curing Age	Specimen	Density (g/cm^3^)
1 day	PC-D1	2.10
	PC-D2	2.11
	PC-D3	2.10
3 days	PC-D4	2.11
	PC-D5	2.12
	PC-D6	2.11
7 days	PC-D7	2.11
	PC-D8	2.12
	PC-D9	2.11
28 days	PC-D10	2.11
	PC-D11	2.12
	PC-D12	2.11
Average	-	2.11

SD/CV: All ages SD = 0.006 g/cm^3^, CV ≤ 0.3%. See Section 3.5.

**Table 14:** Apparent Porosity Results.

Curing Age	Specimen	Porosity (%)
1 day	PC-D1	0.18
	PC-D2	0.16
	PC-D3	0.17
3 days	PC-D4	0.15
	PC-D5	0.14
	PC-D6	0.16
7 days	PC-D7	0.14
	PC-D8	0.15
	PC-D9	0.13
28 days	PC-D10	0.13
	PC-D11	0.14
	PC-D12	0.13
Average	-	0.15

SD/CV: 1-day (0.010%, 5.9%); 3-day (0.010%, 6.7%); 7-day (0.010%, 7.1%); 28-day (0.006%, 4.3%). Full discussion in Section 3.5.

**Table 15:** Safety Factor Assessment—HMA (Flexible) Pavement (PC Patch, Patch-Only Region).

Scenario	Traditional Patch Max PS (MPa)	PC Patch Max PS (MPa)	PC SF Tensile	Tensile Risk	SF Interface (0.78 MPa)	Interface Risk	PC SF Compress.
250-C	0.0967	1.6501	4.62	LOW	0.47	DEBONDING	24.1
500-A	0.1591	0.6006	12.70	LOW	1.30	HIGH	33.2
500-B	0.1935	0.7005	10.89	LOW	1.11	HIGH	45.7
500-C	0.1409	0.8498	8.98	LOW	0.92	DEBONDING	31.7
500-D	0.1649	0.7888	9.67	LOW	0.99	DEBONDING	44.9
1000-A	0.1722	0.6475	11.78	LOW	1.2	HIGH	47.0
1000-B	0.2470	0.7788	9.80	LOW	1.0	HIGH	47.2
1000-C	0.1988	0.8917	8.56	LOW	0.87	DEBONDING	44.2
1000-D	0.1887	0.8060	9.47	LOW	0.97	DEBONDING	38.5

MPS = Maximum Principal Stress at 2 mm from peak stress location. SF Tensile = *f*_t/MPS (*f*_t = 7.63 MPa, 3-day). SF Interface = *f*_bond/MPS (*f*_bond = 0.78 MPa [36]; conservative lower-bound estimate). SF Compress = *f*_c/σ_VM (*f*_c = 85.97 MPa). Risk: FAILED/DEBONDING = SF < 1.0; HIGH = 1.0–2.0; MEDIUM = 2.0–4.0; LOW = >4.0.

**Table 17:** Total System Deformation Comparison-Full Model (mm).

Scenario	HMA + HMA Deform. (mm)	HMA + PC Deform. (mm)	JPCP + Concrete Deform. (mm)	JPCP + PC Deform. (mm)	PC Reduction on HMA (%)
250-C	0.23009	0.21182	0.06782	0.06853	7.9%
500-A	0.23526	0.22520	0.06909	0.06950	4.3%
500-B	0.24994	0.21716	0.06917	0.06913	13.1%
500-C	0.23077	0.20215	0.06786	0.06907	12.4%
500-D	0.23131	0.20712	0.06815	0.06923	10.5%
1000-A	0.25491	0.22797	0.07067	0.07071	10.6%
1000-B	0.25166	0.21482	0.06943	0.07026	14.6%
1000-C	0.23001	0.19478	0.06780	0.06926	15.3%
1000-D	0.23204	0.20519	0.06935	0.07057	11.6%

Values represent maximum total deformation in the full pavement model. Reduction (%) = (HMA_Patch − PC_Patch)/HMA_Patch × 100. Positive values indicate PC reduces system deformation.

**Table 18:** Static vs. Dynamic Safety Factor Comparison—HMA (Flexible) Pavement, PC Patch Only (DIF = 1.30).

Scenario	SF Tensile Static	SF Tensile Dynamic	Dynamic Tensile Risk	SF Int. Static (0.78 MPa)	SF Int. Dyn. (0.78 MPa)	Dyn. Risk (Int.)	SF Compress. Static	SF Compress. Dynamic
HMA (Flexible) Pavement + PC Patch								
250-C	4.62	3.56	MEDIUM	0.47	0.36	DEBONDING	24.1	18.6
500-A	12.70	9.77	LOW	1.30	1.00	DEBONDING	33.2	25.6
500-B	10.89	8.38	LOW	1.11	0.86	DEBONDING	45.7	35.2
500-C	8.98	6.91	LOW	0.92	0.71	DEBONDING	31.7	24.4
500-D	9.67	7.44	LOW	0.99	0.76	DEBONDING	44.9	34.5
1000-A	11.78	9.06	LOW	1.20	0.93	DEBONDING	47.0	36.2
1000-B	9.80	7.54	LOW	1.00	0.77	DEBONDING	47.2	36.3
1000-C	8.56	6.58	LOW	0.87	0.67	DEBONDING	44.2	34.0
1000-D	9.47	7.28	LOW	0.97	0.74	DEBONDING	38.5	29.6

Dynamic SF = Static SF/DIF (DIF = 1.30. PC bulk tensile = 7.63 MPa (3-day); PC-HMA bond = 0.78 MPa (conservative lower-bound estimate). Dyn. Risk = risk classification based on dynamic SF value.

**Table 19:** Static vs. Dynamic Safety Factor Comparison—JPCP (Rigid) Pavement, PC Patch Only (DIF = 1.30).

Scenario	SF Tensile Static	SF Tensile Dynamic	Dynamic Tensile Risk	SF Int. Static (2.82 MPa)	SF Int. Dyn. (2.82 MPa)	Dyn. Risk (Int.)	SF Compress. Static	SF Compress. Dynamic
JPCP (Rigid) Pavement + PC Patch								
250-C	78.05	60.04	LOW	28.85	22.19	LOW	143.2	110.2
500-A	75.74	58.26	LOW	27.99	21.53	LOW	72.1	55.5
500-B	60.01	46.16	LOW	22.18	17.06	LOW	100.2	77.1
500-C	67.20	51.69	LOW	24.84	19.11	LOW	184.9	142.3
500-D	84.37	64.90	LOW	31.18	23.99	LOW	72.6	55.9
1000-A	69.20	53.23	LOW	25.58	19.67	LOW	178.5	137.3
1000-B	60.58	46.60	LOW	22.39	17.22	LOW	160.5	123.5
1000-C	68.36	52.58	LOW	25.26	19.43	LOW	184.4	141.9
1000-D	71.21	54.78	LOW	26.32	20.24	LOW	100.0	76.9

Dynamic SF = Static SF/DIF (DIF = 1.30). PC bulk tensile = 7.63 MPa (3-day); PC-JPCP bond = 2.82 MPa. Dyn. Risk = risk classification based on dynamic SF value. All JPCP scenarios show LOW risk across all checks.

**Table 20:** Interfacial Safety Factor at DIF = 2.00 (95th-percentile spatial-repeatability envelope) for HMA and JPCP partial-depth PC patches.

Scenario	HMA DIF = 2.00 (PC-HMA, 0.78 MPa)	JPCP DIF = 2.00 (PC-JPCP, 2.82 MPa)
250-C	0.23 (DEBONDING)	14.43 (LOW)
500-A	0.65 (DEBONDING)	13.99 (LOW)
500-B	0.56 (DEBONDING)	11.09 (LOW)
500-C	0.46 (DEBONDING)	12.42 (LOW)
500-D	0.49 (DEBONDING)	15.59 (LOW)
1000-A	0.60 (DEBONDING)	12.79 (LOW)
1000-B	0.50 (DEBONDING)	11.20 (LOW)
1000-C	0.43 (DEBONDING)	12.63 (LOW)
1000-D	0.48 (DEBONDING)	13.16 (LOW)

Risk classification: SF < 1.0 → DEB (debonding); SF > 4.0 → LOW. Tensile and compressive safety factors remain in the LOW classification across all scenarios at DIF = 2.00 for both pavement types.

## Data Availability

The datasets generated and/or analyzed during the current study are not publicly available at this stage, as the underlying data are shared across multiple related manuscripts currently in preparation. The data will be made available in a public repository upon the completion and formal publication of the associated studies. In the meantime, the data supporting the reported results are available from the corresponding author upon reasonable request.

## Abbreviations

The following abbreviations are used in this manuscript:

AASHO	American Association of State Highway Officials
AASHTO	American Association of State Highway and Transportation Officials
ABAQUS	Finite Element Analysis Software (Dassault Systèmes)
ACI	American Concrete Institute
ANSYS	Engineering Simulation Software (Ansys, Inc., Canonsburg, PA, USA)
ASTM	American Society for Testing and Materials
CoNap	Cobalt Naphthenate
CTE	Coefficient of Thermal Expansion
CV	Coefficient of Variation
DIF	Dynamic Impact Factor
EDS	Energy-Dispersive X-ray Spectroscopy
EN	European Standard (Norme Européenne)
FDOT	Florida Department of Transportation
FEA	Finite Element Analysis
FHWA	Federal Highway Administration
FWD	Falling Weight Deflectometer
HMA	Hot Mix Asphalt
ITZ	Interfacial Transition Zone
JPCP	Jointed Plain Concrete Pavement
LRFD	Load and Resistance Factor Design
LTE	Load Transfer Efficiency
MEKP	Methyl Ethyl Ketone Peroxide
MPC	Multi-Point Constraint
MPS	Maximum Principal Stress
PC	Polymer Concrete
PCC	Portland Cement Concrete
PDR	Partial-Depth Repair
PIARC	World Road Association (Permanent International Association of Road Congresses)
PPC	Polyester Polymer Concrete
SADT	Self-Accelerating Decomposition Temperature
SD	Standard Deviation
SEM	Scanning Electron Microscopy
SF	Safety Factor
TxDOT	Texas Department of Transportation
UPPC	Unsaturated Polyester Polymer Concrete
UPR	Unsaturated Polyester Resin
UPR-PC	Unsaturated Polyester Resin Polymer Concrete
VOC	Volatile Organic Compounds
WSDOT	Washington State Department of Transportation
